# Development and Implementation of a Pediatric Palliative Care Program in a Developing Country

**DOI:** 10.3389/fpubh.2018.00106

**Published:** 2018-04-16

**Authors:** Megan Doherty, Chloé Thabet

**Affiliations:** ^1^Palliative Care Program, Children’s Hospital of Eastern Ontario, Ottawa, ON, Canada; ^2^University of Ottawa, Ottawa, ON, Canada; ^3^Children’s Palliative Care Initiative in Bangladesh, World Child Cancer, London, United Kingdom

**Keywords:** palliative care, hospices, Bangladesh, children, developing countries, pilot project, patient comfort, neoplasm

## Abstract

**Background:**

Palliative care is recognized as an important component of care for children with cancer and other life-limiting conditions. In resource limited settings, palliative care is a key component of care for children with cancer and other life-limiting conditions. Globally, 98% of children who need palliative care live in low- or middle-income countries, where there are very few palliative care services available. There is limited evidence describing the practical considerations for the development and implementation of sustainable and cost-effective palliative care services in developing countries.

**Objectives:**

Our aim is to describe the key considerations and initiatives that were successful in planning and implementing a hospital-based pediatric palliative care service specifically designed for a resource-limited setting.

**Setting:**

Bangabandu Sheikh Mujib Medical University (BSMMU) is a tertiary referral hospital in Bangladesh. Local palliative care services are very limited and focused on adult patients. In partnership with World Child Cancer, a project establishing a pediatric palliative care service was developed for children with cancer at BSMMU.

**Results:**

We describe four key elements which were crucial for the success of this program: (1) raising awareness and sensitizing hospital administrators and clinical staff about pediatric palliative care; (2) providing education and training on pediatric palliative care for clinical staff; (3) forming a pediatric palliative care team; and (4) collecting data to characterize the need for pediatric palliative care.

**Conclusion:**

This model of a hospital-based pediatric palliative care service can be replicated in other resource-limited settings and can be expanded to include children with other life-limiting conditions. The development of pilot programs can generate interest among local physicians to become trained in pediatric palliative care and can be used to advocate for the palliative care needs of children.

## Introduction

Every year 200,000 children living in low-or middle-income countries (LMIC) will develop cancer (1). In high-income countries, more than 80% of children with cancer will be cured, while in LMIC, only 25% will survive (1). Palliative care can relieve suffering and improve quality of life for children and their families. The International Society of Pediatric Oncology (SIOP) has recommended that palliative care can be considered a key component of the care of all children with cancer in low-income settings and the World Health Organization (WHO) recommends that all hospitals which treat patients with cancer should have a palliative care service (2, 3).

In addition to cancer, children with neonatal conditions, congenital anomalies, HIV/AIDS, and other chronic diseases can benefit from palliative care provided alone or in combination with potentially curative treatments (3, 4). More than 98% of children who need palliative care live in low- or middle-income countries where access to palliative care and pain relief are often extremely limited (3). Recently, a Lancet Commission report proposed an Essential Package of Palliative and Pain Relief Health Services, describing an affordable basic package of medications, medical equipment, and human resources which could alleviate the vast majority of physical and psychological suffering (5).

The WHO has proposed a public health strategy to translate palliative care knowledge into interventions which can reach all children with life-threatening or life-limiting conditions (6). This strategy includes the inclusion of palliative care into all levels of the healthcare system (7). Studies from high-income countries suggest that hospital-based palliative care services improve symptom control and reduce non-beneficial or harmful treatments, such as chemotherapy near the end of life (8–10).

In several low- and middle-income countries, cost-effective community-based palliative care programs have been implemented, however, there is limited evidence to guide clinicians about how to develop hospital-based services, and developing evidence about how to successfully implement hospital-based services will create opportunities for more children in resource-limited settings to access palliative care services (11–14).

## Background and Rationale

### Health Care and Cancer-Related Care in Bangladesh

Bangladesh is a LMIC with a population over 160 million (15). Thirty percent of the population is under 14 years of age (15). The country has made remarkable progress in improving the health of the population, through improvements in maternal and child health. Non-communicable diseases are now a major cause of morbidity and mortality, accounting for 59% of all deaths in 2014 (16). Healthcare expenditures in Bangladesh remain low at US$16.20 per capita, with 64% of this spending being out-of-pocket (17).

Bangladesh is estimated to have 6,000–9,000 new cases of childhood cancer annually, although fewer than 25% of these children are actually diagnosed (18). For the limited number of children who begin treatment, cure rates are reported to be 50–60% (18). Due to financial difficulties and misperceptions about the incurability of cancer, 43% of children diagnosed with cancer do not start treatment or stop treatment prematurely (18). Additionally, due to late diagnosis and advanced disease at presentation, more than 20% of children are incurable at the time of diagnosis and 10% die in early treatment phases (18).

There are an estimated 29,000 children needing palliative care at the end-of-life annually, including children with cancer and other life-threatening or life-limiting conditions (3). There are very few palliative care services operating and most are focused on adult patients (13). Only one pediatric palliative care service exists: Ashic Palliative Care Unit, a privately funded free-standing palliative care facility, which has nine beds. Many more palliative care facilities and services are needed to address the burden of suffering for children with life-limiting conditions and their families in Bangladesh.

## Setting

### Bangabandu Sheikh Mujib Medical University (BSMMU) Resources and Healthcare Statistics

Bangabandu Sheikh Mujib Medical University is a large tertiary specialty referral hospital in Dhaka, Bangladesh. BSMMU provides medical care for thousands of children with life-threatening or life-limiting illnesses annually. It is the largest pediatric oncology center in Bangladesh, where more than 450 patients are diagnosed with cancer annually.

The Department of Pediatric Hematology and Oncology at BSMMU consists of a 31-bed inpatient unit and a large outpatient department, which provides chemotherapy and supportive cancer care 6 days per week. There are no intensive care facilities for children at BSMMU. Most hematology and biochemistry laboratory tests and imaging studies including X-ray, ultrasonography, CT scan, and MRI are available. Patients are charged a nominal fee for BSMMU clinical services, but must pay most of the cost of medical equipment (e.g., IV cannula, IV fluid, urinary catheter) and medications including chemotherapy. Our previous experience found that there is high mortality among children who are diagnosed with cancer at BSMMU due to refusal to begin treatment, abandonment of treatment at an early phase, late or delayed diagnosis, and advanced disease at presentation (18). BSMMU serves as a referral hospital for the entire country, and many children who are diagnosed at this facility are not from Dhaka. As a result, it is common for parents to refuse to start cancer treatment as this would mean relocating to Dhaka, which is financially prohibitive for many families.

A palliative care service, which specifically focuses on the needs of adults with chronic progressive conditions (e.g., advanced cancer, dementia, heart failure, kidney disease), was initiated at BSMMU in 2007. In 2013, this service expanded to become the Centre for Palliative Care, an inpatient and outpatient department for adults with cancer and other serious illnesses. The Centre for Palliative Care has been working with adult medicine specialties to increase awareness about palliative care and provide education through presentation for these departments and divisions, as well as providing clinical rotations in palliative medicine for medical residents (i.e., post-graduate trainee physicians), nurses, social workers, community health workers, and volunteers. These palliative care services are largely focused on adult patients and are not resourced or equipped to meet the unique needs of children and their families. There is a recognized need for a pediatric palliative care service which would focus on the needs of children and their families and would be seamlessly integrated with existing pediatric cancer care services at BSMMU, and which could support children and families throughout their illness trajectory, whether the outcome is cure or death.

Since 2013, the Department of Pediatric Hematology and Oncology at BSMMU has participated in a twinning project with the University College of London and British Columbia Children’s Hospital, facilitated by World Child Cancer. Within this twinning project, the project leaders identified the need for pediatric palliative care and as a result began planning to implement a pediatric palliative care consultation service, with the support of the international twinning partners in 2014. The aim was to develop a sustainable and cost-effective palliative care service while gathering more detailed information about the palliative care needs of children at BSMMU.

In this project, children with cancer were selected as a model for demonstrating a pediatric palliative care service. We chose to focus primarily on these children due to the large number of children with cancer at BSMMU, the strong interest of the oncology clinicians to develop palliative care, and the support of the twinning partnership to provide funding and expertise. We envisaged that this service could be expanded to include children with other life-threatening or life-limiting conditions as the program’s resources and expertise increased in the future.

We chose to initiate a hospital-based palliative care consultation service, given the evidence that this can improve the management of pain and other symptoms, which were observed to be especially problematic for many children with cancer at BSMMU. Additionally, given the significant number of children with cancer and the shortage of inpatient oncology beds, we hoped that a hospital service may reduce length of stay in hospital and relieve suffering by reducing the use of non-beneficial or harmful treatments for children who were dying. Additionally, there are four other government funded hospitals in Bangladesh with specialized pediatric oncology departments, where we hoped that a similar approach could be employed.

In this report, we aim to describe the key considerations and activities which we undertook to develop and implement the pediatric palliative care consultation service at BSMMU, a tertiary hospital in a resource limited setting. During this project, our goal was to develop a sustainable and cost-effective palliative care service for children at one of the largest government hospitals treating children in Bangladesh, while gathering information to quantify the need for these services. We hope that our experience can help others to develop pediatric palliative care services in other settings with similar resource limitations.

## Developing and Implimenting a Pediatric Palliative Care Service

This is the first study to describe the development of a pediatric palliative care consultation service for hospital inpatients and outpatients in Bangladesh. The four key areas of action for this project were:

1. Raising awareness and sensitization
2. Education and training
3. Implementing a pediatric palliative care service
4. Collecting data to provide a detailed picture of the palliative care needs of children and families at BSMMU.

### Raising Awareness and Sensitization

Early in the planning phase, we identified the lack of knowledge about the philosophy of palliative care and misperceptions about palliative care as major barriers which could limit the access of patients to palliative care services and had the potential to restrict our abilities to develop the service. This lack of awareness was a problem both for health administrators within the hospital and for healthcare practitioners.

Senior members of our palliative care planning team met with key hospital administrators to describe how palliative care could help patients and their families, discussing the goals of reducing suffering and enhancing quality of life for patients. Additionally, we provided information about the significant need for pediatric palliative care at BSMMU and sought to gain their support for the implementation of a pediatric palliative care service. We met with influential senior physicians, who were determined with key health opinion leaders in the medical community, to gain their support for our plans. At a national pediatric oncology workshop, a member of the team gave a plenary address about how palliative care could help to reduce suffering for children and families.

As we came closer to launching the palliative care service, we developed and implemented activities to sensitize the staff in pediatric oncology to the philosophy and practices of palliative care. To reach the maximum number of individuals, we spoke at education seminars, provided training workshops on pain management and palliative care, and distributed promotional materials. Once the pediatric palliative care team had been formed, the team regularly attended bedside clinical rounds to increase the awareness and visibility of the team and build trust between the teams which allowed for timely referrals.

### Providing Education and Training

Training activities were primarily focused on clinicians working in the pediatric oncology department. We first met with oncology clinicians to identify their key educational needs in palliative care. Together, our teams developed the core competencies for oncology clinicians in palliative care, which included the following:

1. Understand the philosophy and practice of pediatric palliative care, especially as this relates to pediatric oncology
2. Know how to assess pain in children of a variety of ages, including infants and children with a decreased level of consciousness
3. Know how to treat pain, with an emphasis on the WHO pain ladder for children
4. Describe how to appropriately use cancer directed therapies, especially when treating children who cannot be cured
5. Communicate effectively with families, in the setting of discussions about prognosis, relapse, and end of life issues.

Many clinicians specifically requested training communicating “bad news” to families, so the palliative care team developed an interactive communication workshop to improve skills in effectively and compassionately delivering bad news. Training was provided to oncology clinical staff on a regular basis and in a variety of formats, including continuing professional development workshops and hospital scientific seminars. The palliative care team also provided informal teaching through clinical interactions during ward rounds. Trainee physicians and nurses, who rotate through the pediatric oncology department, were also provided with basic education sessions about palliative care and pain management.

We also developed posters, pocket-reference cards, and handouts on the following topics:

1. Procedural pain management
2. How to apply of topical anesthetic cream
3. Pain assessment tools for children
4. Non-pharmacological pain management techniques
5. Pharmacological pain treatments
6. Management of common physical symptoms
7. Requirements for prescribing controlled medicines (e.g., morphine).

**Figure 1:**
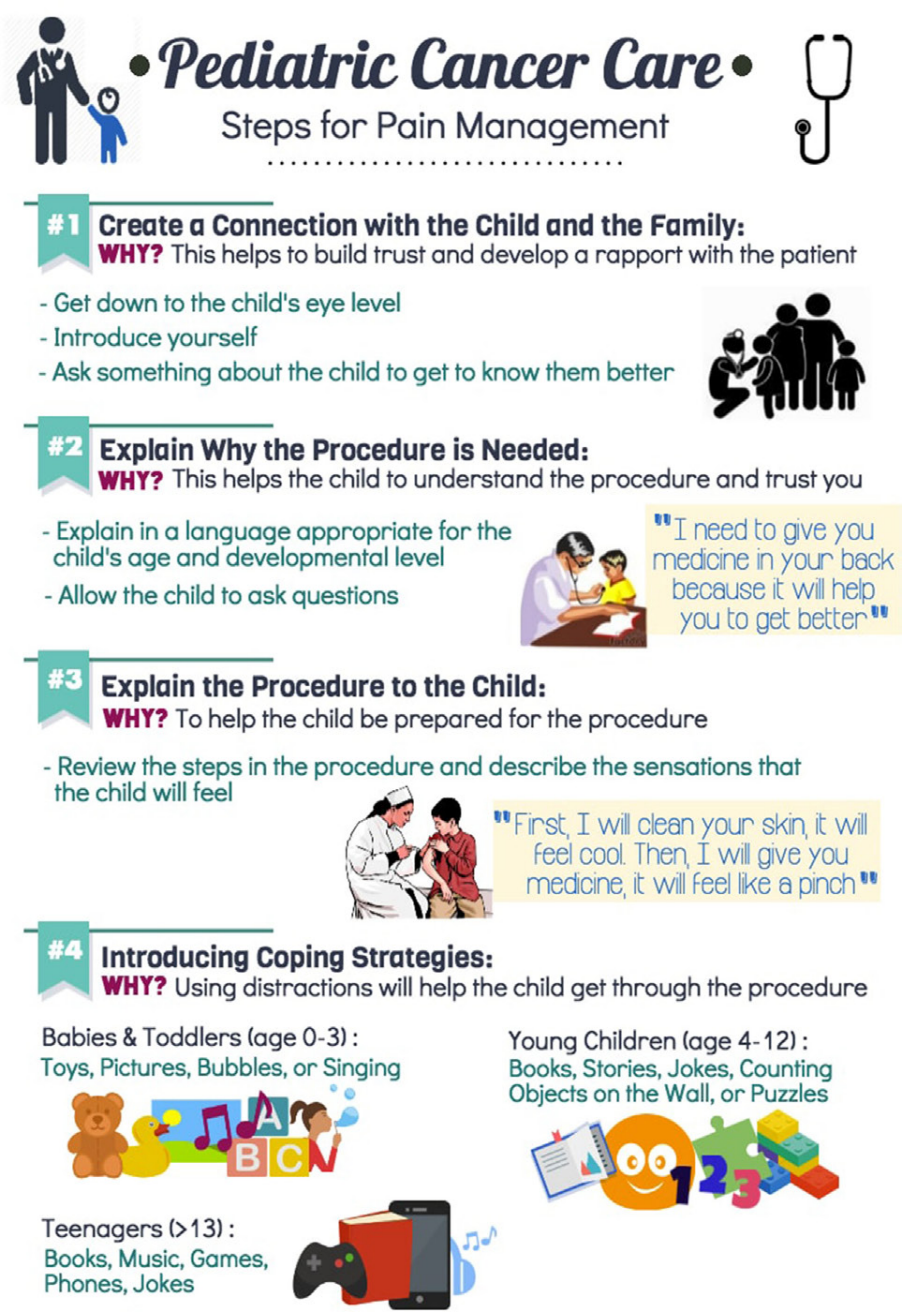
Pediatric cancer care. Steps for pain management.

**Figure 2:**
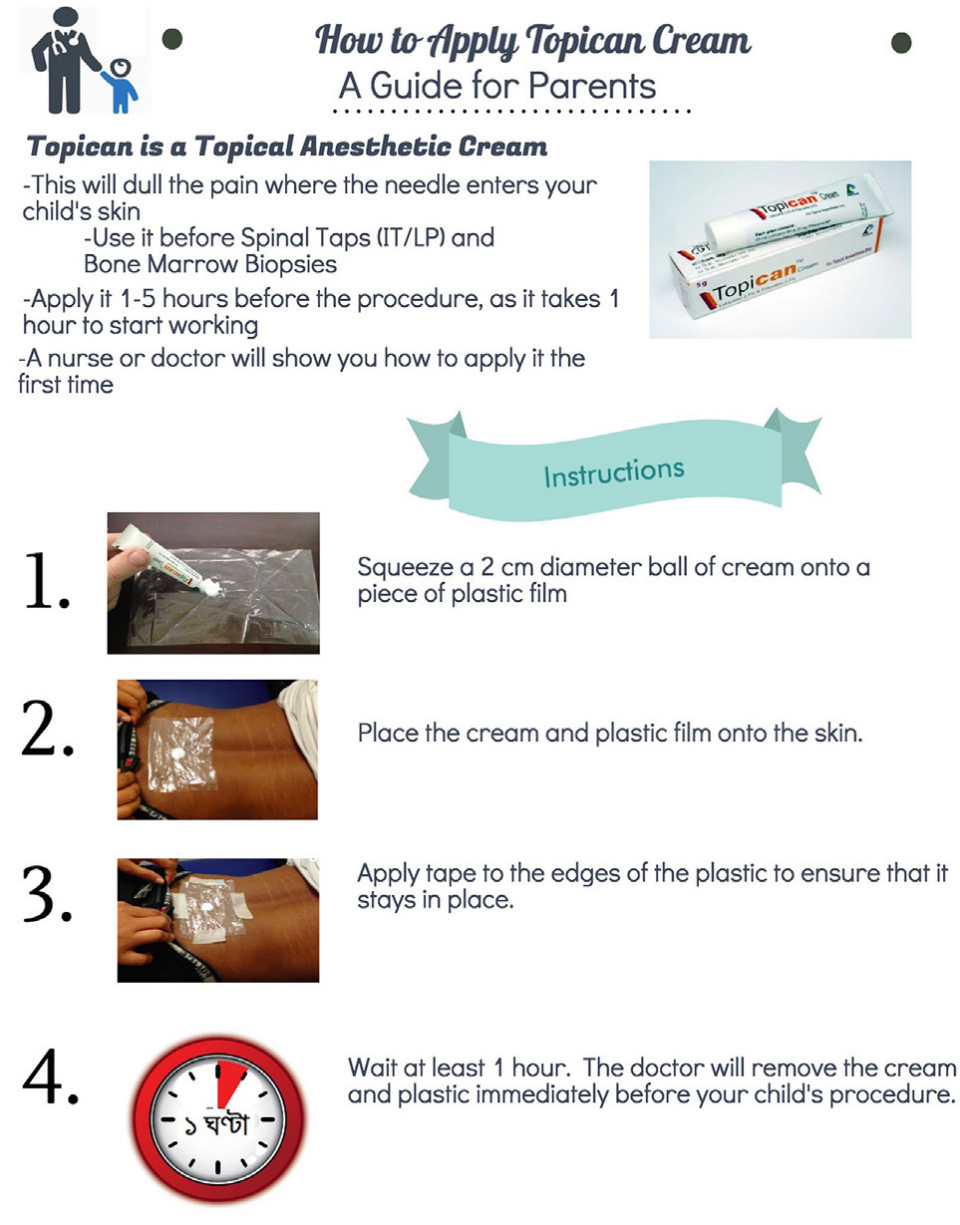
How to apply Topican cream. A guide for parents.

These materials were designed to re-enforce the concepts discussed in training events and lead to greater awareness of these topics among oncology clinicians. We were careful to ensure that the educational materials which we developed used simple and inexpensive medicines and medical equipment which are readily available in Bangladesh. Figures 1 and 2 shows several examples of the pain treatment posters which were developed for use on the oncology ward.

### Implementing a Pediatric Palliative Care Service

In January 2014, a pediatric palliative care team was formed, consisting of a pediatric palliative care specialist consultant and a medical officer as well as a team of trained psychosocial support volunteers. The pediatric palliative care team provided consultations for inpatients and outpatients, coordinated the transition from curative to palliative-focused care, facilitated home-based care, and provided ongoing education and support for oncology clinicians and a 24-h phone service for patients and families. Due to financial issues and a shortage of allied healthcare workers, it was not possible to recruit a nurse or social worker. To address issues of financial burden, during this pilot project, oral morphine was provided free of cost for all oncology patients, through funding from World Child Cancer.

The team provided home visits, if the patient resided in Dhaka, but in most cases, patients lived greater than several hours’ journey from Dhaka, and the team was only able to provide telephone-based support. All patients who are discharged from hospital were encouraged to find a local physician who could provide care in coordination with the pediatric palliative care team, but in our experience many families struggled to find a doctor who was willing to do this.

A group of dedicated volunteers was recruited and trained to provide play, art, and music care for children who were admitted to the oncology inpatient ward. A playroom was constructed within the ward. The volunteers supported the emotional and social needs of children and their caregivers through the use of play, art, and music. Parents and nurses consistently reported that the children were happier and less withdrawn after having been to the playroom. In cases where a child was not able to leave their bed, a volunteer would provide age appropriate toys and activities with the child at his or her bedside.

### Data Collection

Data was collected using Pond4Kids,^1^ an online pediatric oncology database. Routine collection of demographic data about all oncology patients had been going on since 2013. We recorded clinical information about symptoms, their treatment, and psychosocial and counseling support which was provided using Pond4Kids. Data collection was done daily by a member of the palliative care team, as part of the course of regular charting activities. The team found that this was an efficient and effective way to record standardized clinical information and often referred to this information during subsequent visits, since it was difficult to find the relevant information in the hospital medical records. Later, the team used this data to provide hospital administrators with clear information about the palliative care needs of children with cancer.

## Discussion

In this report, we describe the key steps and practical considerations to develop and implement a pediatric palliative care consultation service at a tertiary hospital in a resource limited setting in Dhaka, Bangladesh. The crucial aspects of this pilot project included raising awareness among hospital administrators and clinical staff, providing education and training for hospital staff, implementing a clinical palliative care service, and collecting data to define the palliative care needs of children with cancer. The use of volunteers to provide play, art, and music was a simple and effective way to expand the palliative care supports in which this small pediatric palliative care team was able to provide. We will discuss the practical implications, lessons learned, and limitations of our approach under the four key aspects of the project.

### Raising Awareness and Sensitization

In Bangladesh, palliative care has not been incorporated into the undergraduate curriculum for medicine or nursing, and as a result there is limited awareness among clinicians about palliative care or knowledge that palliative care should be a core component of routine health care. Our initial activities to build awareness about palliative care among hospital administrators and front-line healthcare workers were implemented to address this issue and were important to ensure support for our project.

When speaking about palliative care, we found that it was effective to share qualitative information (e.g., a compelling story about an individual child whose suffering could have been relieved with palliative care), in addition to providing specific information about the number of children needing palliative care. With this approach, we were able to evoke compassion, but we could also use the data to have a rigorous discussion about the exact number of children in need. Quantitative data is necessary for healthcare administrators to make decisions about the planning and implementing of healthcare services, but a compelling story can be a source of motivation for change. We often began our discussion by inviting administrators and clinicians share the story of a family member or close friends who had suffered with cancer or another serious illness. We observed that asking them to share these stories helped them to better understand palliative care and allowed us gain insight into their perspectives and experiences.

Previous studies have identified physician reluctance to refer children to palliative care as a significant barrier to the provision of pediatric palliative care (19, 20). Initially, we designed our awareness and training events to address the reasons for this reluctance, including discomfort about discussing death with patients and families, and the idea that palliative care may “feel like giving up.” However, most oncology physicians at BSMMU were enthusiastic about palliative care and as soon as the palliative care service became available, began to refer children. Other authors have identified a shortage of time as a significant barrier to the provision of palliative care. In our situation, due to the high volume of patients, oncology physicians find it especially difficult when patients and families needed counseling (e.g., cancer which was not curable or relapsed). The pediatric palliative care team could spend more time providing additional counseling and support to patients and families in these situations, which meant that the team was embraced quickly by the oncology clinicians.

### Education and Training

Lack of training of healthcare professionals (HCP) in LMIC has been identified as a major obstacle to the provision of palliative care (21). We developed and provided palliative care training for healthcare providers, with particular attention to providing content that was practical and could be directly applied in Bangladesh. Our team investigated the local pharmaceutical market to find out which symptom control medications were the least expensive and readily available. The team then focused on the use of these medicines in our clinical practice and in training sessions.

Twinning projects, often incorporate visits from clinicians from the high-income partner to provide training and mentorship. However, it is important to be cautious in the use of training from foreign visiting experts, since the recommendations can be difficult for local staff to implement if content has not been adapted to the local resource constraints and may be impractical to implement (22).

It is important to assess the effects of training and adapt the training to meet desired objectives (22). For example, during the planning phase, working with oncology clinicians, we identified that they wanted to be able to prescribe morphine for children who had moderate or severe pain. We then provided training sessions about how to assess pain in children and how to safely use morphine. However, despite this training, we found that physicians were still not prescribing morphine. We investigated further and discovered that physicians were unaware of the information which was legally required on a morphine prescription and thus were not providing appropriate prescriptions to patients. Subsequently, we incorporated this information into training sessions and developed a pocket reference card which specified which information was required. This lead to an increase in the number of children being appropriately treated with morphine. This also illustrates the need to examine all potential barriers to change when attempting to implement a new clinical practice.

Initially, there was opposition to providing printed materials during training from senior oncology clinicians, as they feared that those attending the sessions would simply discard the materials after the training. However, though experience, we found that education sessions were more effective when we provided handouts and pocket reference card. Participants reported that these allow them to understand the training better, review what they had learned at a later date, and incorporate the teaching into their clinical practice.

Traditionally, education in Bangladesh has been didactic and focused on knowledge acquisition. We found that healthcare professionals were unfamiliar with interactive lecturing techniques and struggled at first to participate in group discussions and role play sessions. Through our experiences we found it helpful to orient learners to this teaching style and we began incorporating a short introduction session before any training to explain to learners that we would be expecting them to participate in discussion and small group activities and how this would improve their learning. When using role-play to teach communication skills, we allocated additional time for explaining and demonstrating this technique before inviting learners to participate, since they had rarely observed this type of activity before.

We found that trainers also needed training in the principles of adult learning to increase the efficacy of the training they provided. We developed a professional development training workshop which addressed this knowledge and skills gap among trainers. This workshop explained the core principles of adult learning (i.e., learner centered, problem-based, active learning, giving formative feedback, supportive learning environment) and provided practical advice about interactive teaching techniques (e.g., case discussions, questioning the audience, role-playing, small group discussions) and presentation skills.

### Implementing a Pediatric Palliative Care Service

The implementation of clinical palliative care services helped to reinforce the concept of palliative care as a key component of the health care for children with cancer among clinical staff in oncology. By directly observing the palliative care service, staff could appreciate the benefits that this had for patients and families and they become keen to integrate palliative care into the care of all children with cancer.

We were unable to incorporate a psychosocial support worker to the palliative care team due to a shortage of these professionals at the hospital, which meant that no one from BSMMU could be assigned this role. Our experience is that funding agencies are frequently unwilling to support staff salaries, which limited our ability to have a social worker or counselor hired for the palliative care team.

A lack of time, was another barrier encountered by the palliative care team, as the team found they did not have sufficient time to support all of the children being treated in the oncology department (inpatient and outpatient departments) who could have benefited from their services. The team chose to focus the majority of their time on the children admitted to the inpatient ward.

### Data Collection

There is a lack of high quality data about the need for palliative care for children (4). We collected data about the symptoms of children with cancer and the palliative care services which they needed, including pain treatment, other symptom treatments, counseling, and psychosocial support. We found that measuring the needs of children was important so that this information could be used to advocate for expansion of palliative care services at BSMMU. The results of the needs identified by this project will be reported elsewhere.

The development of this project was instrumental in generating interest among local physicians to study palliative care. Since the implementation of this service, three pediatric oncology physicians from Bangladesh have completed a 1-month basic certificate course in pediatric palliative care in Hyderabad, India. The experience from this project also directly led to the development of the Children’s Palliative Care Initiative in Bangladesh,^2^ a larger project focused on improving pediatric palliative care services for children across Bangladesh. This project involved awareness raising efforts on a larger scale, with the general public, government officials, and at other hospitals as well as educational workshops at hospitals across Bangladesh and the development of a community-based palliative care service in an urban informal settlement (Korail Slum) in Dhaka, Bangladesh. The results of this work will be reported elsewhere.

## Ethics Statement

This study was carried out in accordance with the recommendations of WMA Declaration of Helsinki. The protocol was approved by the Institutional Review Board of Bangabandu Sheikh Mujib Medical University, approval number BSMMU/2016/344.

## Data Management and Sharing

Pond4Kids (www.pond4kids.org) was used to collect demographic data about all children with cancer who are diagnosed at BSMMU.

## Author Contributions

All authors have approved this version of the article for publication. MD participated in all phases of this study and article development, including developing the concept for this work, acquisition, analysis, and interpretation of data, drafting and revision of the article. CT participated in analysis, and interpretation of data and drafting and revision of the article.

## Conflict of Interest Statement

The authors declare that the research was conducted in the absence of any commercial or financial relationships that could be construed as a potential conflict of interest.

## Footnotes

^1^www.pond4kids.org (Accessed: January 17, 2017).

^2^https://childrenspalliativec.wixsite.com/cpcib (Accessed: January 08, 2017).

## References

[1] Kellie SJ Howard SC. Global child health priorities: what role for paediatric oncologists? Eur J Cancer (2008) 44(16):2388–96. 10.1016/j.ejca.2008.07.022 18799306

[2] WHO. Planning and Implementing Palliative Care Services: A Guide for Programme Managers [cited January 8, 2018]. Available from: http://apps.who.int/iris/bitstream/10665/250584/1/9789241565417-eng.pdf.

[3] WHO. Global Atlas of Palliative Care at the End of Life (2014). Available from: http://www.who.int/nmh/Global_Atlas_of_Palliative_Care.pdf.

[4] Connor SR Downing J Marston J. Estimating the global need for palliative care for children: a cross-sectional analysis. J Pain Symptom Manage (2017) 53(2):171–7. 10.1016/j.jpainsymman.2016.08.020 27765706

[5] Alleviating the Access Abyss in Palliative Care and Pain Relief – An Imperative of Universal Health Coverage: the Lancet Commission Report [cited December 8, 2017]. Available from: http://www.thelancet.com/commissions/palliative-care. 10.1016/S0140-6736(17)32513-8 29032993

[6] Weltgesundheitsorganisation, editor. Cancer Pain Relief and Palliative Care: Report of a WHO Expert Committee (Technical Report Series). Geneva: World Health Organization (1990). 75 p. 1702248

[7] Stjernswärd J Foley KM Ferris FD. The public health strategy for palliative care. J Pain Symptom Manage (2007) 33(5):486–93. 10.1016/j.jpainsymman.2007.02.016 17482035

[8] Higginson IJ Finlay I Goodwin DM Cook AM Hood K Edwards AGK Do hospital-based palliative teams improve care for patients or families at the end of life? J Pain Symptom Manage (2002) 23(2):96–106. 10.1016/S0885-3924(01)00406-7 11844629

[9] O’Mahony S Blank AE Zallman L Selwyn PA. The benefits of a hospital-based inpatient palliative care consultation service: preliminary outcome data. J Palliat Med (2005) 8(5):1033–9. 10.1089/jpm.2005.8.1033 16238516

[10] Penrod JD Deb P Luhrs C Dellenbaugh C Zhu CW Hochman T Cost and utilization outcomes of patients receiving hospital-based palliative care consultation. J Palliat Med (2006) 9(4):855–60. 10.1089/jpm.2006.9.855 16910799

[11] Ddungu H Palliative care: what approaches are suitable in developing countries? Review. Br J Haematol (2011) 154(6):728–35. 10.1111/j.1365-2141.2011.08764.x 21707576

[12] Caruso Brown AE Howard SC Baker JN Ribeiro RC Lam CG. Reported availability and gaps of pediatric palliative care in low- and middle-income countries: a systematic review of published data. J Palliat Med (2014) 17(12):1369–83. 10.1089/jpm.2014.0095 25225748 PMC4268583

[13] Lynch T Connor S Clark D. Mapping levels of palliative care development: a global update. J Pain Symptom Manage (2013) 45(6):1094–106. 10.1016/j.jpainsymman.2012.05.011 23017628

[14] Downing J Powell RA Marston J Huwa C Chandra L Garchakova A Children’s palliative care in low-and middle-income countries. Arch Dis Child (2015) 101(1):85–90. 10.1136/archdischild-2015-308307 26369576

[15] World Bank. Bangladesh | Data [cited June 10, 2016]. Available from: http://data.worldbank.org/country/bangladesh.

[16] World Health Organisation. Cancer. Noncommunicable Diseases (NCD) Country Profiles, 2014 (2016). Available from: http://www.who.int/nmh/countries/bgd_en.pdf.

[17] World Health Organization. Regional Office for the Western Pacific. Bangladesh Health System Review. Geneva, Switzerland: World Health Organization (2015).

[18] Islam A Eden T. Brief report on pediatric oncology in Bangladesh. South Asian J Cancer (2013) 2(2):105–6. 10.4103/2278-330X.110516 24455571 PMC3876649

[19] Ahmad NU Khan F Quadir SS Rahman M Laskar MH Akhtaruzzaman AKM Conceptual prevalence in palliative care amongst the physicians of Bangabandhu Sheikh Mujib Medical University: a comparison between the post-graduate trainees and the trainers. J Bangladesh Soc Anaesthesiol (2014) 22(1):26–31. 10.3329/jbsa.v22i1.18098

[20] Knapp C Thompson L Factors Associated with Perceived Barriers to Pediatric Palliative Care: A Survey of Pediatricians in Florida and CaliforniaPalliative Medicine (2012) [cited June 28, 2017]. Available from: http://journals.sagepub.com/doi/abs/10.1177/0269216311409085. 10.1177/0269216311409085 21680751

[21] Hannon B Zimmermann C Knaul FM Powell RA Mwangi-Powell FN Rodin G. Provision of palliative care in low- and middle-income countries: overcoming obstacles for effective treatment delivery. J Clin Oncol (2016) 34(1):62–8. 10.1200/JCO.2015.62.1615 26578612

[22] Hopkins J Burns E Eden T International twinning partnerships: an effective method of improving diagnosis, treatment and care for children with cancer in low-middle income countries. J Cancer Policy (2013) 1(1):e8–19. 10.1016/j.jcpo.2013.06.001

